# Morphological Characteristics of the Thymus and Spleen and the Subpopulation Composition of Lymphocytes in Peripheral Blood during Systemic Inflammatory Response in Male Rats with Different Resistance to Hypoxia

**DOI:** 10.1155/2019/7584685

**Published:** 2019-04-01

**Authors:** Dzhuliia Sh. Dzhalilova, Anna M. Kosyreva, Mikhail E. Diatroptov, Natalia A. Zolotova, Ivan S. Tsvetkov, Vladimir A. Mkhitarov, Olga V. Makarova, Dmitry N. Khochanskiy

**Affiliations:** Department of Immunomorphology of Inflammation, Federal State Budgetary Scientific Institution “Research Institute of Human Morphology,” Tsyurupy St., 3, Moscow, Russia

## Abstract

On the model of the systemic inflammatory response (SIRS), induced by lipopolysaccharide (LPS), the morphological and functional changes in the thymus and spleen and the subpopulation composition of peripheral blood lymphocytes of rats differing in resistance to hypoxia were studied. It was demonstrated that the level of endotoxin in blood serum after 3 hours of LPS administration in susceptible-to-hypoxia rats was 64 times higher than in the control group, while in tolerant-to-hypoxia animals it was only 8 times higher in 6 hours. After 24 hours of LPS injection, only in susceptible-to-hypoxia rats did the level of C-reactive protein in blood serum increase. There is a difference in the dynamics of morphological changes of lymphoid organs after LPS injection in tolerant- and susceptible-to-hypoxia animals. After 3 hours of LPS administration, the tolerant-to-hypoxia rats showed no changes in the thymus, spleen, and subpopulation composition of lymphocytes in peripheral blood. After 6 hours there was only a decrease in B-lymphocytes and increase in cytotoxic T-lymphocytes and NK cells. After 1 day of LPS injection, the tolerant-to-hypoxia rats had devastation in PALS of the spleen. After 3 hours of LPS injection the susceptible-to-hypoxia animals had reactive changes in the lymphoid organs: decrease of the thymus cortex, narrowing of the marginal zones of spleen lymphoid follicles, widening of their germinal centers, and a decrease in the absolute number of cytotoxic T-lymphocytes, NK cells, and B-lymphocytes. After 24 hours of LPS injection the tolerant-to-hypoxia animals had a greater absolute number of T-lymphocytes and NK cells in comparison with the susceptible rats. Thus, in animals with different resistance to hypoxia the LPS-induced SIRS is characterized by different dynamics of morphological and functional changes of the thymus and spleen. The obtained data will serve as a basis for the development of new individual approaches to the prevention and treatment of infectious and inflammatory diseases.

## 1. Introduction

Many patient-specific factors, in particular age, sex, ethnicity, etc., have an impact on the severity, mortality, and survival rates of infectious and inflammatory diseases, including sepsis [1–3]. Nevertheless, the clinical course, mortality, survival rate, and possibility of development of nosocomial infections may vary even in a population of people of the same age and sex [3]. Since hypoxia develops during inflammation, the individual tolerance to the lack of oxygen may play an important role in the mechanism of resistance to it.

It is known that people and laboratory animals are heterogeneous by the resistance to hypoxia [4–8]. Organisms with various resistance to the lack of oxygen differ in many parameters of integrative systems, such as the central nervous system, the endocrine system, and metabolism (antioxidant activity, mitochondrial enzyme complex I activity, level of norepinephrine, prolactin, corticosterone, etc.) [4, 9], including the content of hypoxia-inducible factor (HIF-1*α*), which is induced by hypoxia [5, 10]. It has been demonstrated that, in susceptible-to-hypoxia rats under the normal conditions, the level of HIF-1*α* in the neocortex is 1.7 times higher than in tolerant rats [5]. We earlier demonstrated the differences in the serum level of 8-isoprostane and the anti-inflammatory cytokine transforming growth factor-*β* (TGF-*β*) content in tolerant- and susceptible-to-hypoxia rats in the early period after the acute hypoxic exposure. Animals with different resistance to hypoxia have various adaptive abilities and predisposition to the development of inflammatory diseases: in susceptible-to-hypoxia animals, the 8-isoprostane level, which is a marker of oxidative stress, increases after hypoxic exposure. It is associated with damage of cellular macromolecules and an increase in the level of TGF-*β* [11].

According to the literature, the processes of hypoxia and inflammation are closely interconnected. HIF-1 is related to the family of transcription factors NF-*κ*B (nuclear factor-kappa B), which is a key mediator of the inflammatory response and controls the expression of the genes of various cytokines, chemokines, acute phase proteins, and adhesion molecules [12]. It was demonstrated that the short-term stay of animals and humans under hypoxic conditions (at an altitude of more than 3,400 m above sea level) leads to an increase in the level of inflammatory markers in the serum: IL-6, IL-6R, and C-reactive protein (CRP) [13]. Oxygen deficiency causes activation of cells of both innate and adaptive immunity, inhibits neutrophil apoptosis [14], increases migration of neutrophils and macrophages [15, 16], and stimulates differentiation of T-helper cells [17] and T-regulatory cells [18]. Based on the current data, the expression of HIF-1 in immune cells is crucial for their functional activity. Increasing its expression contributes to the survival of basophils, eosinophils, neutrophils, and mast cells, stimulates the production of proinflammatory cytokines, and induces polarization of macrophages in the M1 type. HIF-1 increases the cytotoxic activity of CD8+ T-cells and NK cells and impacts the proliferation of B-lymphocytes and T-regulatory cells [19].

According to the current data, HIF can play both proinflammatory and anti-inflammatory roles in different models of inflammatory processes [20]. On the model of acute colitis in mice it was demonstrated that the deficiency of HIF-1*α* correlated with high mortality rates, and the surviving mice showed severe clinical manifestations of colitis [21]. In contrast, in systemic infections, such as sepsis, high level of HIF-1*α* is associated with greater mortality, the increase in the content of proinflammatory cytokines, IL-1*β* and tumor necrosis factor-*α* (TNF-*α*), and the decrease in anti-inflammatory cytokine IL-10, which promotes the development of proinflammatory reactions [22]. Thus, in the literature the question being discussed is whether the increase of HIF-1 level is considered as a potential prognostic marker of sepsis [23].

One of the models of the systemic inflammatory response syndrome (SIRS) is endotoxinemia, caused by the injection of high doses of lipopolysaccharide (LPS), which leads to the development of acute respiratory distress syndrome, increased coagulation with the manifesting of disseminated intravascular coagulation (DIC) syndrome, dystrophic changes, and necrosis in the liver [24–26]. This model is easily reproducible and it was demonstrated earlier that there are sexual differences in the development of SIRS [25]. In the development of SIRS and sepsis, hypoxia, which results from DIC microcirculatory disorders, plays a key role [24]. However, the severity of hypoxic damage of tissues and organs not only depends on microcirculatory disorders, but also mostly is determined by individual resistance of organism to hypoxia. According to our data (unpublished), after 6 hours of LPS injection at a dose of 1.5 mg/kg, in comparison with the tolerant ones, in the susceptible-to-hypoxia rats, the expression level of* Hif-1α* in the liver was 2 times higher. It was combined with the increase in the expression of* Nf-κb *and in the content of the proinflammatory cytokine IL-1*β* in blood serum after 3 and 6 hours of LPS administration. These results suggest that in tolerant- and susceptible-to-hypoxia animals the severity of the inflammatory response may differ. The inflammation is a complex immunologically conditioned response of the organism, and its development in the early stages is realized by the mechanisms of innate immunity. Available data, mentioned above, about the features of the endocrine system reaction, differences in the expression of* Hif-1α*,* Nf-κb*, etc. [4, 5], in rats with different resistance to oxygen deficiency suggest the existence of differences in the reaction of other integrative systems of the organism, including the immune system and closely related inflammatory processes in susceptible- and tolerant-to-hypoxia animals. Understanding the mechanisms of the interrelation between resistance to the lack of oxygen and the peculiarities of immune response may help to develop the approaches to personalized immunomodulatory therapy of infectious and inflammatory diseases among patients with the different hypoxia resistance. However, there are no data in the literature about the differences in the morphological characteristics of the thymus and spleen, as well as the subpopulation composition of peripheral blood lymphocytes during SIRS in animals with different resistance to hypoxia.

Therefore, the aim of the study was to identify differences in the morphological changes of the thymus and spleen and the subpopulation of lymphocytes in peripheral blood at different times after LPS administration in male Wistar rats with different resistance to hypoxia.

## 2. Materials and Methods

### 2.1. Experimental Animals

Male Wistar rats (n=60), 2-3 months old, 220–240 g body weight, were purchased from the Animal Breeding Facility Branch Stolbovaya of the Federal State Budget Institution of Science, the Scientific Center for Biomedical Technologies of the Federal Medical and Biological Agency, Russia. Six rats per cage (18.5 x 60 x 38 cm) were housed in a temperature-regulated room at a 12:12 h light-dark cycle, relative humidity, between 40% and 50%, and unlimited access to water and food (Char, JSC Range-Agro, Russia). The study received permission from the Bioethics Committee of the Science Research Institute of Human Morphology (Protocol No. 16, November 11, 2015). All experimental work involving animals was carried out according to the European Convention for the Protection of Vertebrate Animals used for Experimental and Other Scientific Purposes (1986), and all efforts were made to minimize suffering and distress of animals.

### 2.2. Determination of Resistance to Hypobaric Hypoxia

Hypoxic tolerance was determined by measuring the time taken for the onset of gasping (gasping time). Adult male Wistar rats were exposed, one at a time, to simulated hypobaric hypoxia, equivalent to the altitude of 11,500 m, as described previously [5–9, 11, 27–29] in an animal decompression chamber coupled to a mercury barometer (equivalent to 180 mmHg). All the decompression and recompression instances were achieved gradually at a rate of 600 m (≈40 mmHg)/min to prevent any tissue injury as a result of a sudden fall or rise in ambient pressure. The airflow in the chamber was 2 L/min, and the relative humidity was maintained at 40 to 50 %. The time taken for appearance of the first sign of gasping, a characteristic hyperventilatory response, was recorded using an electronic stopwatch. Based on their gasping time, animals were categorized into three groups: normal (80–240 s, n=12) and tolerant (>240 s, n=25) and susceptible (<80 s, n=23) to hypoxia. Normal rats were not used in the experiments. After the determination of resistance to hypobaric hypoxia, all rats were found to be alive and have resumed normal activity without any evident sign of pathology.

### 2.3. Experimental Model of SIRS

One month after the determination of resistance to hypoxia rats of the experimental groups were injected intraperitoneally with LPS from* E. coli* O26:B6 (Sigma, USA) at a dose of 1.5 mg/kg, leading to pathological changes in target organs [25, 30]. The animals were euthanized with an overdose of carbon dioxide gas using a gradual fill (30% chamber volume per minute) technique after 3 h, 6 h, and 24 hours of LPS injection (5–6 animals for each term). The timing choice was determined by the fact that* Nf-κb* expression increased after 1-2 and 6 hours [31], and pronounced pathological changes in the target organs developed on the 1st day after the administration of LPS [32]. In control groups the tolerant- (n=5) and susceptible- (n=5) to-hypoxia rats received an intraperitoneal injection of physiological saline.

### 2.4. Mortality of Animals from SIRS

Within a day after the introduction of LPS, some of the animals died. Mortality rates of rats in response to the injection of LPS were 3 out of 18 (17%) in the susceptible-to-hypoxia rats and 2 out of 20 (10%) in the tolerant-to-hypoxia ones. The mortality rate, caused by development of endotoxin shock, appeared within 6 hours after the injection of LPS. The choice of LPS dose was considered and approved by the Bioethics Committee of the Science Research Institute of Human Morphology (Protocol No. 16, November 11, 2015).

### 2.5. Sample Collection

Venous blood from jugular veins [33] was centrifuged for 20 minutes at 200 g. The obtained serum was frozen at -70°C and stored for no more than 2 months. The lungs of animals were fixed in Carnoy's solution (60 ml ethanol, 30 ml chloroform, and 10 ml glacial acetic acid) for 2 hours, the thymus and spleen were fixed in Bouin's solution (75 ml picric acid, 25 ml formalin, and 5 ml glacial acetic acid) [34] for 24 hours, and organs were embedded in paraffin according to routine procedures. Histological sections of 4-5 *μ*m thickness were produced and stained with hematoxylin and eosin (BioVitrum, Russia).

### 2.6. Morphological Study

The evaluation of histological slides was randomized and blinded. Using the light microscopic method, the number of neutrophils in the interalveolar septa of the lungs was counted in 10 high-power fields of view (25000 *μ*m^2^) per section, and the average number of neutrophils per slide was determined [25, 35]. In the histological slices of the thymus and spleen, the volume fraction of the functional zones of the immune organs was determined by the point-count method [36].

### 2.7. Enzyme-Linked Immunosorbent Assay (ELISA)

We estimated the concentration of CRP (Cloud-Clone Corp., China) in the serum by ELISA. The endotoxin level in the serum was estimated by the chromogenic Limulus amebocyte lysate (LAL) test (HBT, USA). For determination of the intensity of the color reaction, the microplate analyzer Anthos 2010 (Austria) was used.

### 2.8. Flow Cytometry

Absolute and relative numbers of lymphocytes of various subpopulations in peripheral blood were counted using flow cytometry (Beckman Coulter, USA). The following antibodies (eBioscience, USA) were used for immune phenotypic analysis of the main subpopulations of lymphocytes: anti-rat CD3 (for T-lymphocytes), anti-rat CD4 (for CD3+CD4+ T-helpers), anti-rat CD8a (for CD3+CD8a+ cytotoxic T-cells), anti-rat CD45R (for CD3-CD45R+ B-lymphocytes), anti-mouse/rat Foxp3 and anti-rat CD25 (for CD4+CD25+Foxp3+ regulatory T-cells), and anti-rat CD314 (for CD3-CD314+ natural killer cells). Erythrocytes were lysed with the OptiLyse C solution (eBioscience, USA).

### 2.9. Statistics

Digital data were tested for normality using the Kolmogorov-Smirnov test in Statistica 8.0. To isolate the group or groups that differ from the others, we used the nonparametric Mann-Whitney* U* test and multiple comparison procedure. In cases when* p*<0.05, multiple comparison procedures were performed by the Kruskal-Wallis method. The median and interquartile range (Me, low-high) were calculated for values of the measured parameters. The differences were considered statistically significant when* p*<0.05. Data are represented graphically using box-and-whisker plots, which demonstrate the median, interquartile range, lower extreme (25%), and upper extreme (75%) of the data.

## 3. Results

### 3.1. Determination of Endotoxin and CRP Level

**Figure 1:**
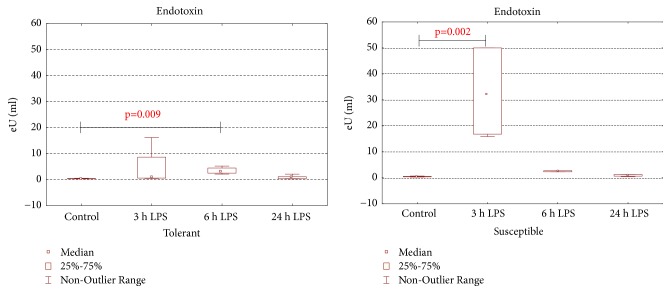
Endotoxin serum levels in tolerant- and susceptible-to-hypoxia rats after 3, 6, and 24 hours of LPS administration. Data are presented as median (25%, 75%); the statistical significance of differences (*p*) is determined by the Kruskal-Wallis method (*n* = 5 in each group).

According to ELISA, the serum level of endotoxin after 3 h of LPS injection in the susceptible-to-hypoxia animals increased compared with the control group and was 36 times higher than in the tolerant ones (*p*=0.03). After 6 hours of LPS administration in susceptible-to-hypoxia animals, endotoxin serum level was normalized, but in tolerant rats it was increased by 8 times compared with the control group (Figure 1). After 24 hours of LPS injection, the serum level of endotoxin did not differ from the control group in both tolerant- and susceptible-to-hypoxia rats.

Only in the susceptible-to-hypoxia rats was the level of CRP in serum increased from 1363 (1128–1551 pg/ml) in the control group to 2421 (1810–2844 pg/ml) after 24 hours of LPS administration (*p* = 0.04). In tolerant-to-hypoxia rats the level of CRP in serum after 24 hours of LPS injection was 2115 (1974–2397 pg/ml) and did not change in comparison with the control group (1833 (1645–2585 pg/ml)). There were no statistically significant differences in the CRP content between tolerant- and susceptible-to-hypoxia animals both in the control group and after 24 hours of LPS administration.

### 3.2. Morphological Changes in the Lungs

**Figure 2:**
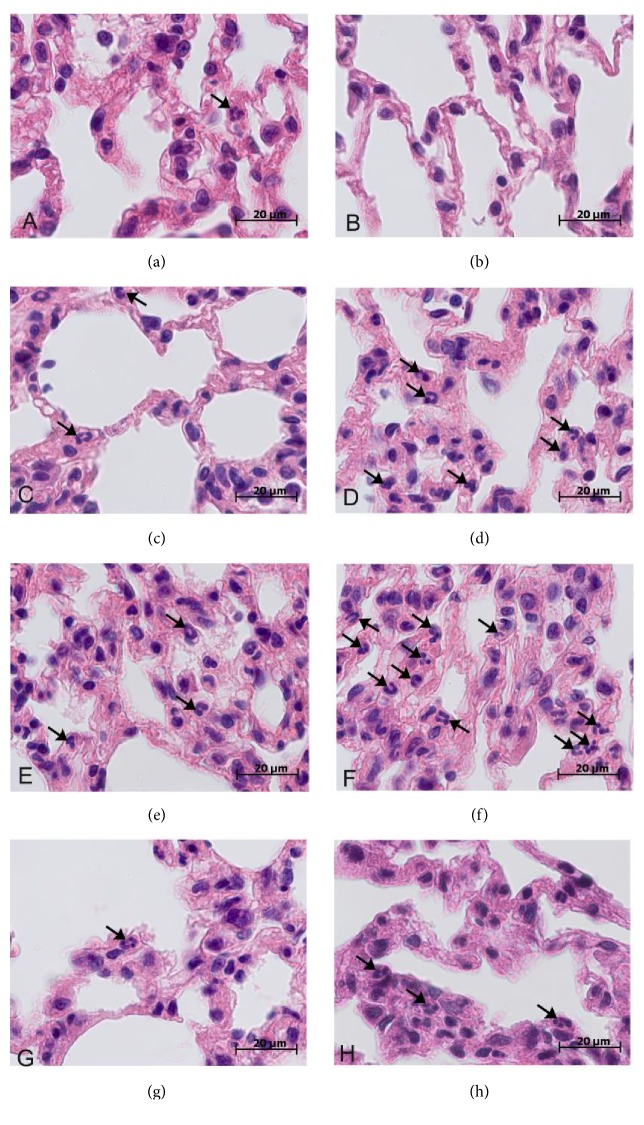
Morphological changes in the lungs of tolerant- and susceptible-to-hypoxia male Wistar rats after 3, 6, and 24 hours of LPS administration. Hematoxylin and eosin staining. Original magnification: ((a)-(h)) 1000x. Neutrophils are marked via arrows. (a) Tolerant-to-hypoxia rat, control group; few neutrophils in the field of view. (b) Susceptible-to-hypoxia rat, control group; thin interalveolar septa without neutrophils in the field of view. (c) Tolerant-to-hypoxia rat, 3 h LPS; interalveolar septa with neutrophils. (d) Susceptible-to-hypoxia rat, 3 h LPS; pronounced neutrophil infiltration in thickened interalveolar septa. (e) Tolerant-to-hypoxia rat, 6 h LPS; mild neutrophil infiltration in thickened interalveolar septa. (f) Susceptible-to-hypoxia rat, 6 h LPS; high number of neutrophils in thickened interalveolar septa. (g) Tolerant-to-hypoxia rat, 24 h LPS; interalveolar septa with few neutrophils. (h) Susceptible-to-hypoxia rat, 24 h LPS; interalveolar septa with neutrophils.

There was no difference in the number of neutrophils in interalveolar septa in the control groups of tolerant- and susceptible-to-hypoxia rats (Table 1). In all periods after the LPS injection, infiltration of interalveolar septa with neutrophils, hyperemia, and intra-alveolar edema was observed in the lungs of tolerant- and susceptible-to-hypoxia animals (Figure 2).

The number of neutrophils in the interalveolar septa was significantly higher in the susceptible-to-hypoxia rats after 6 hours of LPS injection in comparison with the tolerant ones (Table 1). After 24 hours of LPS administration in both susceptible- and tolerant-to-hypoxia animals the number of neutrophils in the interalveolar septa was the same as in the control groups.

### 3.3. Morphological Changes of the Thymus and Spleen

**Figure 3:**
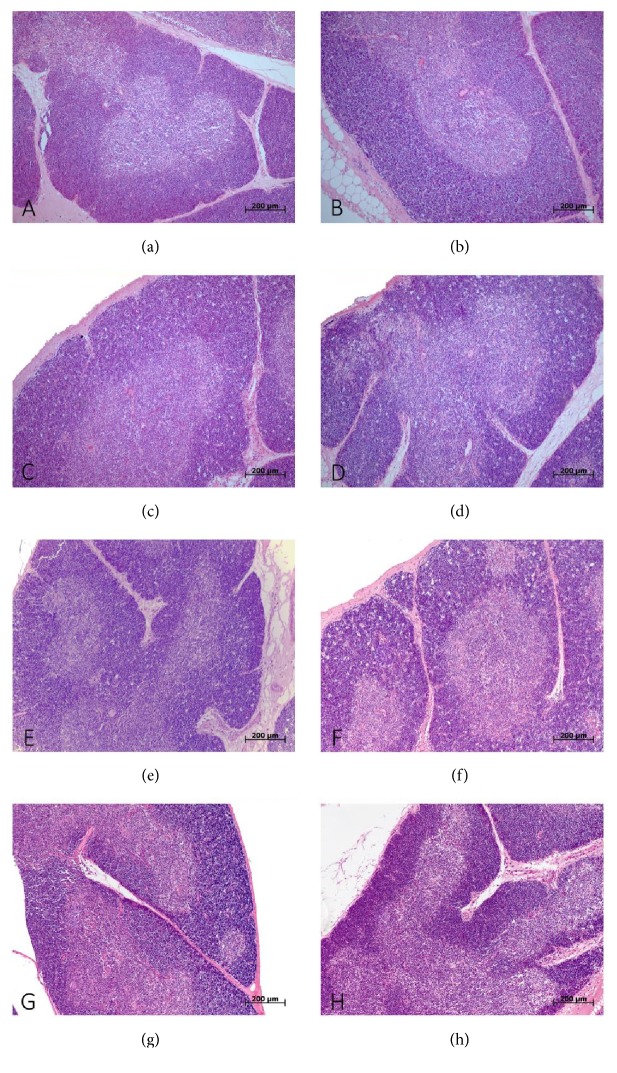
Morphological changes in the thymus of tolerant- and susceptible-to-hypoxia male Wistar rats after 3, 6, and 24 hours of LPS administration. Hematoxylin and eosin staining. Original magnification: 100x. (a) Tolerant-to-hypoxia rat, control group; cortex is wide, and the border between the cortex and medulla is readable. (b) Susceptible-to-hypoxia rat, control group; the proportion of the cortex/medulla is 1:1, and the border between them is readable. (c) Tolerant-to-hypoxia rats, 3 h LPS; narrowing of cortex (with a starry-sky aspect). (d) Susceptible-to-hypoxia rats, 3 h LPS; mild involution, narrowing of cortex, more pronounced compared with tolerant ones. (e) Tolerant-to-hypoxia rats, 6 h LPS; mild involution; the cortex is narrow. (f) Susceptible-to-hypoxia rats, 6 h LPS; a pronounced starry-sky aspect, devastation of cortex, and macrophages and dying lymphocytes. (g) Tolerant-to-hypoxia rats, 24 h LPS; narrow cortex; the border between the cortex and medulla is readable. (h) Susceptible-to-hypoxia rats, 24 h LPS; narrow cortex; the border between the cortex and medulla is readable.

**Figure 4:**
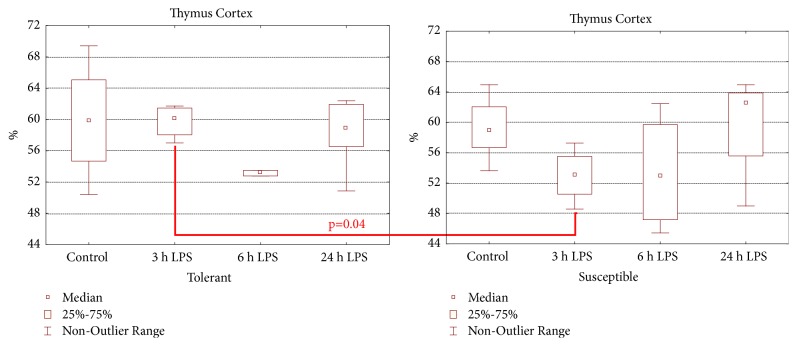
The volume fraction of the thymus cortex in tolerant- and susceptible-to-hypoxia rats after 3, 6, and 24 hours of LPS administration. Data are presented as median (25%, 75%); the statistical significance of differences (*p*) between tolerant and susceptible rats is determined by the Mann-Whitney* U* test and between different time points after LPS injection by the Kruskal-Wallis method (*n* = 5 in each group).

During morphological study in all periods after LPS administration signs of mild involution of the thymus were found in both tolerant- and susceptible-to-hypoxia animals. After 3 and 6 hours of LPS injection the rats with various hypoxia tolerance had a narrowing of the thymus cortex; there were present macrophages and fragments of dying cells. The involution of the thymus was more pronounced after 6 hours of LPS injection (Figure 3). According to morphometric study after 3 hours of LPS administration the volume fraction of the thymus cortex (Figure 4) in susceptible rats (53.1 (50.5–55.5)%) was statistically significantly lower (*p*=0.04) than in the tolerant-to-hypoxia rats (60.2 (58.1–61.5)%).

**Figure 5:**
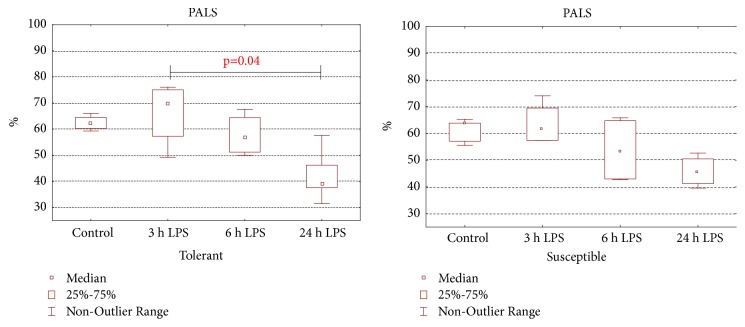
The volume fraction of the spleen PALS in tolerant- and susceptible-to-hypoxia rats after 3, 6, and 24 hours of LPS administration. Data are presented as median (25%, 75%); the statistical significance of differences (*p*) is determined by the Kruskal-Wallis method (*n* = 5 in each group).

According to morphological study in the spleens (see Figure S1 (A–H) for comprehensive image analysis) of rats with various hypoxia resistance after LPS injection, narrowing of PALS (the periarterial lymphatic sheaths) zone and marginal zone of lymphoid follicles and enlargement of lymphoid follicles and germinal centers were observed. According to morphometrical analysis in the spleens only of the susceptible-to-hypoxia rats the volume fraction of white pulp, formed by PALS and lymphoid follicles, was increased in comparison with the control group (35.6 (35.6-36.6)%) after 24 hours of LPS administration (49.3 (43.6–54.0)%),* p*=0.01. After 24 hours of LPS injection in the spleens only of the tolerant-to-hypoxia rats the volume fraction of PALS decreased in comparison with the corresponding parameter after 3 hours (Figure 5).

**Figure 6:**
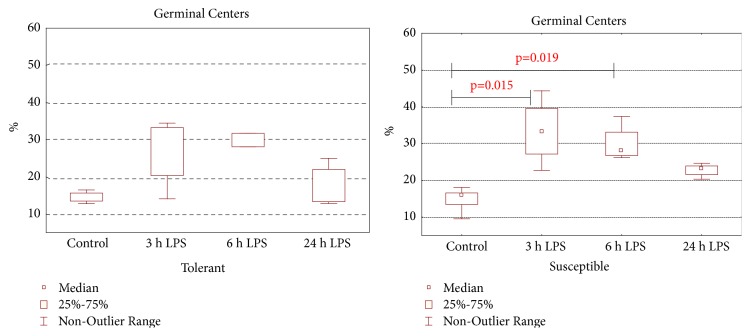
The volume fraction of germinal centers of spleen lymphoid follicles in tolerant- and susceptible-to-hypoxia rats after 3, 6, and 24 hours of LPS administration. Data are presented as median (25%, 75%); the statistical significance of differences (*p*) is determined by the Kruskal-Wallis method (*n* = 5 in each group).

In comparison to the control groups, both in susceptible- (36.7 (36.1–42.9)%) and in tolerant-to-hypoxia rats (37.9 (35.5–39.8)%) after 24 hours of LPS administration, the volume fraction of lymphoid follicles of the spleen was expanded to 54.4 (49.5–58.7)% and 61.1 (53.8–62.4)%, respectively (*p* = 0.014). In the control groups of tolerant- and susceptible-to-hypoxia rats, the volume fraction of germinal centers did not differ. After 3 and 6 hours of LPS injection, only in susceptible-to-hypoxia rats was there an expansion of the germinal centers (Figure 6).

**Figure 7:**
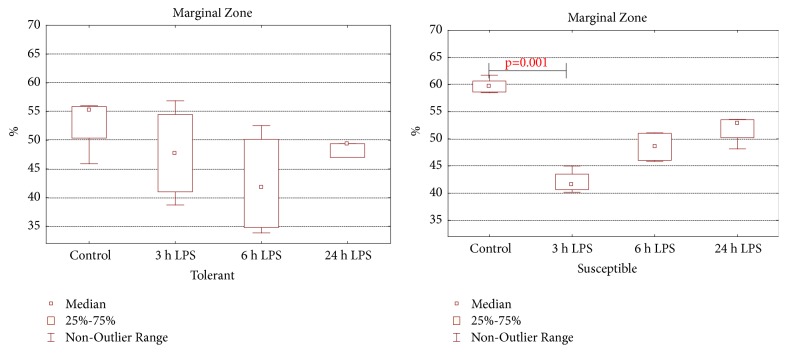
The volume fraction of the marginal zone of spleen lymphoid follicles in tolerant- and susceptible-to-hypoxia rats after 3, 6, and 24 hours of LPS administration. Data are presented as median (25%, 75%); the statistical significance of differences (*p*) is determined by the Kruskal-Wallis method (*n* = 5 in each group).

In comparison with the tolerant ones (55.3 (50.4–55.9)%), in susceptible-to-hypoxia rats of the control group (59.6 (58.6–60.6)%), the volume fraction of the marginal zone was higher (*p* = 0.014). Only in susceptible-to-hypoxia animals did the marginal zone narrow after 3 hours of LPS administration (Figure 7). After 6 and 24 hours of LPS injection the volume fraction of the marginal zone of lymphoid follicles normalized.

Thus, after LPS injection the tolerant-to-hypoxia rats had devastation of the T-dependent zone, while the susceptible animals had activation of the B-zone.

### 3.4. Subpopulation of Lymphocytes in Peripheral Blood

**Figure 8:**
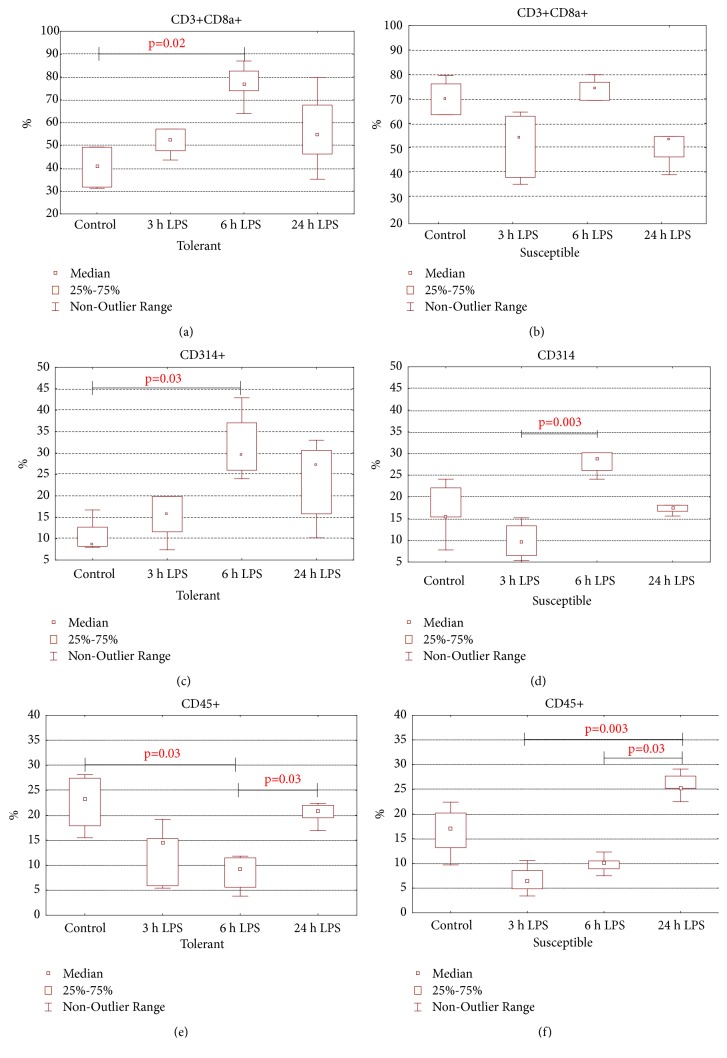
The relative number of ((a), (b)) cytotoxic T-lymphocytes (CD3+CD8a+), ((c), (d)) NK cells (CD314+), and ((e), (f)) B-lymphocytes (CD45+) in peripheral blood of tolerant- (a, c, and e) and susceptible- (b, d, and f) to-hypoxia rats after 3, 6, and 24 hours of LPS administration. Data are presented as median (25%, 75%); the statistical significance of differences (*p*) is determined by the Kruskal-Wallis method (*n* = 5 in each group).

With flow cytometry it was demonstrated that the absolute number of cytotoxic T-lymphocytes and NK cells in the peripheral blood of tolerant-to-hypoxia rats of the control group was significantly lower than in susceptible-to-hypoxia animals. After 3 hours of LPS injection, only in susceptible-to-hypoxia rats did the absolute number of cytotoxic T-lymphocytes, B-lymphocytes, and NK cells significantly decrease (Table 2). The relative number of cytotoxic T-lymphocytes (Figure 8(a)) and NK cells (Figure 8(c)) in peripheral blood of tolerant-to-hypoxia rats significantly increased after 6 hours, but the relative number of B-lymphocytes (Figure 8(e)) and T-helpers and the absolute number of T-regulatory cells decreased (Tables 2 and 3). In susceptible rats, the absolute number of T-helpers and T-regulatory cells was significantly decreased after 6 hours of LPS administration (Table 2), and the relative number of NK cells increased (Figure 8(d)). After 24 hours of LPS injection, the parameters normalized in both tolerant- and susceptible-to-hypoxia rats, but in the tolerant animals the absolute number of T-lymphocytes and NK cells was significantly higher, and the relative number of B-lymphocytes was lower than in the susceptible-to-hypoxia ones.

## 4. Discussion

It is known that the severity of infectious and inflammatory diseases, including sepsis, mortality, and survival rates, depends on many patient-specific factors [1–3]. It is probably due to individual sensitivity to hypoxia, because during inflammation there is a disturbance in function of blood microcirculation, which leads to hypoxia affecting immune cells.

In experimental studies, intraperitoneal administration of various doses of endotoxin, such as* E. coli* LPS, is used to model SIRS and sepsis [25, 37–39]. Based on the previous researches, it can be mentioned that high concentration of endotoxin in serum may lead to undesirable circumstances, such as the development of septic shock. If it is possible to eliminate the endotoxin, the survival rate will demonstrate the positive tendency to avoid the multiple organ failure [40, 41].

It is known that when LPS is introduced, the activation of toll-like receptors (TLR4) occurs, which leads to the induction of NF-kB expression. NF-kB promotes the production of proinflammatory cytokines, IL-1*β*, TNF-*α*, and IL-6, and the initiation of an immune response [24, 31, 42]. Based on our data, susceptible-to-hypoxia rats had a multiple increase in endotoxin level in serum after 3 h of LPS administration, which, apparently, causes a more pronounced inflammatory response: previously we have shown (unpublished data) that, after 3 and 6 hours of LPS injection at a dose of 1.5 mg/kg, the increase in* Nf-κb* expression in the liver accompanied by an increase in serum IL-1*β* concentration is observed only in susceptible-to-hypoxia rats, which indicates the development of more pronounced LPS-induced inflammation in the livers of these animals.

Endotoxin blood level depends on the balance of its intestine absorption and elimination. There are some ways to neutralize LPS: different LPS-binding proteins, lipoproteins that quickly bind LPS in plasma and do not allow it to interact with TLR4; deacetylation, dephosphorylation of LPS, phagocytosis by Kupffer cells in the liver, rapidly removing LPS from the blood, which then enters the hepatocytes [43–45]. In addition, LPS can be recognized by the TLR4 receptors of neutrophils and macrophages and internalized by phagocytosis, which is accompanied by pronounced proinflammatory responses. The more pronounced increase of endotoxin in susceptible-to-hypoxia rats is evidence for the predominance of its absorption over elimination.

In addition, one of the indicators of SIRS severity is the increased concentration of CRP. As the level of CRP synthesis reflects the intensity of the inflammatory process, it is used as one of the clinical markers of infectious and inflammatory diseases, including sepsis [46, 47]. It was demonstrated that high concentrations of CRP in the development of sepsis indicate unfavorable prognosis [48]. According to the literature, the main inducer of CRP gene expression is IL-6, with IL-1 enhancing the effect during the acute phase of an inflammatory/infectious process [49]. However, although IL-6 is necessary for CRP gene induction, it is not sufficient to achieve this alone [50]. The stimulation of CRP synthesis in the liver mainly occurs in response to proinflammatory cytokines, which are synthesized under the influence of NF-kB [51], most notably IL-6 and to a lesser degree IL-1 and TNF-*α* [52]. CRP activates NF-kB [53] and stimulates additional synthesis of proapoptotic cytokines and inflammatory mediators include IL-1*β*, TNF-*α*, and reactive oxygen species [54, 55].

In response to the injection of LPS, the level of CRP rises significantly after 10–12 hours and reaches the maximum after 24–48 hours [56–59]. Based on our results, the significant increase in CRP level in serum after 24 hours occurred only in susceptible-to-hypoxia rats, not in tolerant rats. Apparently, the high level of the* Nf-κb* expression in the livers of only susceptible-to-hypoxia rats contributes the increases of IL-1*β* level after 3 hours of LPS administration at a dose of 1.5 mg/kg. Since, according to data [60], injection of LPS induces IL-6 synthesis, it is likely that its level in susceptible-to-hypoxia rats also increases. IL-6 can activate CRP; an additional increase in IL-1*β*, apparently, causes activation of the synthesis of CRP in susceptible-to-hypoxia rats. The additional effect of CRP on proinflammatory cytokines leads to the development of a more pronounced inflammatory reaction in response to the administration of LPS in susceptible-to-hypoxia rats and a less pronounced reaction in tolerant ones.

The LPS-induced increase of endotoxin level and CRP in susceptible-to-hypoxia rats is followed by pronounced neutrophilic infiltration of interalveolar septa in the lungs, which points to the development of a more pronounced inflammatory response in these animals. Larger numbers of neutrophils in the lungs of susceptible-to-hypoxia rats may be due to the high level of NF-kB-dependent production of chemokines and adhesion molecules [12].

Therefore, the LPS-induced inflammatory response is more pronounced in susceptible-to-hypoxia rats, appearing as an elevated level of endotoxin, CRP, and an inflammatory reaction in the lungs.

Hypoxia is an important physiological stimulus for organisms. HIF-1 is a heterodimeric complex, consisting of two subunits, HIF-1*α* and HIF-1*β* [61]. With sufficient oxygen content, HIF-1*α* under the influence of inhibitors is destroyed in proteasomes, and HIF-1*β* is constitutively present in cells. Under hypoxic conditions, HIF-1*α* accumulates in the cytoplasm, translocates into the nucleus, forming a complex with HIF-1*β*, and binds the HREs (hypoxia-response elements) on the promoters of hypoxia-responsive genes, inducing their expression [62, 63]. Under the regulation of HIF-1 are many genes, responsible for the reaction for the hypoxic influence, including the genes of glucose transporters, erythropoietin, and vascular endothelial growth factor (VEGF). According to the published data, hypoxia contributes to the development of the inflammatory processes, such as the signal pathways, which are activated after the hypoxic influences and are closely connected with inflammation [64–66]. HIF-1 is associated with the nuclear factor regulating the processes of inflammation, NF-*κ*B. It is known that inhibitors that promote ubiquitin-dependent destruction of HIF-1*α* also control the activity of the kinase complex, which is responsible for the regulation of NF-*κ*B activity [64, 67]. The proximal part of the promoter of the HIF-1*α* gene contains the NF-*κ*B binding site [68–70]. S. Frede et al. in 2006 [71] revealed that LPS can induce the NF-*κ*B-dependent increase in mRNA and HIF-1*α* protein levels. If HIF-1 is activated by hypoxia, the transcription of various targeted genes is enhanced, which allows the organism to adapt to the lack of oxygen. During the induction through the NF-*κ*B-dependent pathway, the genes of proinflammatory cytokines are activated [72]. Hyperactivation of the production of inflammatory molecules leads to a disturbance of hemodynamics, activation of coagulation with the increase of NO synthesis, vasoconstriction, and the development of hypoxia, which also has effects on the immune cells [73]. During the SIRS, both hypoxic conditions arising due to microcirculatory disorders and factors of the inflammatory environment may contribute to the increase in HIF-1 expression. In our unpublished study, after 6 hours of LPS injection, the level of liver* Hif-1α* expression increased significantly in both susceptible- and tolerant-to-hypoxia rats; however, in susceptible rats, the increase was twice higher than in tolerant rats. Nevertheless, in tolerant-to-hypoxia rats there was no increase in the level of expression of* Nf-κb*, which indicates that in these animals HIF-1*α* is activated independently from the NF-*κ*B pathway and does not lead to a pronounced inflammatory reaction. Increasing the expression of HIF-1 in different pathways in tolerant- and susceptible-to-hypoxia rats can cause significant differences in inflammation in SIRS and is also accompanied by the development of immune response of various severity and direction.

In our work, the more pronounced thymus involution with narrowing of the cortex was observed in susceptible-to-hypoxia animals after 3 hours of LPS administration, in comparison with tolerant rats. It is known that LPS has a direct negative effect on thymus cells, in particular, on double positive lymphocytes (CD4+CD8+), inhabiting the cortex [25, 74], and proinflammatory cytokines cause thymus involution in various infections [75, 76]. Probably more pronounced thymus involution in susceptible-to-hypoxia rats is due to high endotoxin and proinflammatory cytokines levels, as well as due to activation of T-lymphocyte migration from the thymus to the spleen and other organs of the peripheral immune system, which explains the absence of changes in the volume fraction of the PALS of the spleens in susceptible-to-hypoxia rats. The reduction of the PALS of the spleen occurred only in tolerant-to-hypoxia rats, which indirectly indicates the activation of the cellular reaction of the immune response.

Morphological changes in the spleen, detected in the early stages of development of SIRS in susceptible-to-hypoxia rats, reflect the activation of proliferation and migration of B-lymphocytes. It is known that LPS activates the proliferation of B-lymphocytes, the switching of antibody classes, and differentiation into plasma cells [77]. As a result of antigenic stimulation, the marginal zone reduces due to the migration of lymphocytes to lymphoid follicles and redistribution of dendritic cells in T-zones [78, 79], as well as the expansion of germinal centers due to the activation of proliferation and differentiation of B-lymphocytes. The growth of the volume fraction of white pulp was also observed after 24 hours of LPS administration, which is consistent with literature data for mice [78, 80]. In contrast to follicular B-lymphocytes, B-cells of the marginal zone are activated more rapidly by stimulating LPS without interaction with T-cells and differentiating into Ig-secreting cells [81, 82]. B-cells of the marginal zone and dendritic cells, capturing the antigen from the vessels of red pulp, migrate from the marginal zone of the lymphoid follicles for subsequent antigen presentation to lymphocytes [83]. More pronounced changes in the B-zone of the spleen after the introduction of LPS were detected in susceptible-to-hypoxia rats, which, apparently, is associated with activation of the humoral immunity.

According to our data, in the susceptible-to-hypoxia rats of the control group, the marginal zone was much wider than in tolerant animals which could indirectly indicate the higher antibody production level in these rats. At the same time, the level of endotoxin was increased to the greater extent in susceptible-to-hypoxia rats after 3 hours of LPS administration and normalized after 6 hours. Besides the abovementioned ways of endotoxin elimination it is also neutralized by antibodies [84]. IgM and IgG can bind and neutralize endotoxin, and, what is more, they can also activate the classical complement pathway. According to the literature, the first wave of low-affinity antibodies, which play an important role in the early elimination of pathogens, is secreted by B-cells of the marginal zone of the spleen in response to mitogens such as LPS [85–87]. Apparently, in susceptible-to-hypoxia rats, antibodies synthesized by the lymphocytes of the splenic marginal zone cause rapid elimination of endotoxin from the blood.

Moreover, except for the activation of B-lymphocytes, under the influence of LPS both CD4+ and CD8+ T-lymphocytes proliferate and secrete Th1-type cytokines [88]. It has been demonstrated that, in mice, LPS stimulates CD8+ T-lymphocytes that are capable of suppressing the humoral immune response to bacterial lipopolysaccharides such as type III pneumococcal polysaccharide [89]. According to our data, only in tolerant-to-hypoxia rats after 6 hours of LPS administration was there the increase in the relative number of cytotoxic T-lymphocytes in the peripheral blood. We have discovered that only in susceptible-to-hypoxia rats was the decrease in the number of CD3+CD8+ T-lymphocytes observed. According to the literature, the decrease in the number of cytotoxic T-lymphocytes correlates with the severity of abdominal sepsis in mice [90]. The pronounced decrease in cytotoxic T-lymphocytes in patients with sepsis evidences immunosuppression and also increases the possibility of secondary infection [91, 92].

In our work, the increase in the relative number of NK cells was demonstrated in the tolerant-to-hypoxia rats after 6 hours of LPS administration compared with the control group, and after 24 hours their absolute content was higher in tolerant rats. The role of NK cells is actively studied at the present time and according to the literature, the main function of NK cells in various models of infectious diseases is the contact cytolysis, as well as the synthesis of IFN*γ* [93, 94]. At the same time, the disruption of the function of NK cells weakens the response to infection [95]. The injection of LPS leads to a decrease in NK cells and CD8+ T-lymphocytes only in susceptible-to-hypoxia rats, which, on the one hand, may indicate a more pronounced migration of CD3+CD8+ and CD314+ lymphocytes into target organs for LPS, followed by activation of proinflammatory reactions in them, and, on the other hand, may indicate pronounced apoptosis of cytotoxic T-lymphocytes and NK cells, which can lead to suppression of the immune response [96]. On the contrary, after LPS administration in tolerant-to-hypoxia animals, the absolute number of T-lymphocytes and NK cells was significantly higher than in susceptible rats, indicating the activation of cell-mediated immunity.

As it was stated before, the growth in the expression level of HIF-1 can be both proinflammatory (LPS-induced sepsis model) and anti-inflammatory (colitis model). It is defined by the conditions of inflammation. Earlier we demonstrated that increased expression of HIF-1*α* in systemic infections leads to a greater severity of the inflammatory response in susceptible-to-hypoxia animals. The data of the present research broaden the knowledge about the connection between resistance to hypoxia and HIF-1*α* expression level and features of immune response during SIRS. However, further studies are essential for the understanding of the possible protective role of increased expression of HIF-1*α* in susceptible-to-hypoxia animals on a colitis model.

As mentioned above, the immune system reactions in animals with different tolerance to hypoxia are not studied sufficiently. We showed that there is predominant activation of cellular immunity in tolerant-to-hypoxia rats, while susceptible animals have a predominance of humoral immunity. These results may be applied for developing new approaches to personalized immunomodulatory therapy with regard to hypoxia tolerance. As the effectiveness of immunomodulatory drugs may not be the same in individuals with different resistance to hypoxia, it is likely that the dose and course of treatment with immunomodulatory drugs should be selected taking into consideration the individual resistance to hypoxia and the possibility of various reaction of the immune system, which was demonstrated in the present research. Therefore, our data probably will allow developing new approaches for personalized medicine, taking into consideration the individual's initial resistance to hypoxia and the features of inflammatory and immune response.

## 5. Conclusions

Thus, for the first time it has been demonstrated that tolerant- and susceptible-to-hypoxia rats are characterized by a different severity of inflammation and reactions of the immune system in response to the administration of LPS. Susceptible-to-hypoxia rats are characterized by a more pronounced LPS-induced inflammatory response, which is revealed in a greater number of neutrophils in the interalveolar septa of the lungs and an increased content of serum CRP and endotoxin. Compared with tolerant animals, susceptible rats have a more pronounced reduction of the thymus cortex, as well as activation of the spleen B-zone, an increase in the number of B-lymphocytes in the peripheral blood, which in sum indicates the activation of the humoral reaction of the immune response. In tolerant-to-hypoxia rats, on the contrary, in the spleen the T-dependent PALS narrows, and the number of cytotoxic T-lymphocytes and NK cells increases in peripheral blood, which indicates the activation of the cell-mediated immunity. The revealed features of inflammatory reactions in animals with different resistance to hypoxia will serve as a basis for the development of new individual approaches to the prevention and treatment of infectious and inflammatory diseases.

## Figures and Tables

**Table 1:** The number of neutrophils in the interalveolar septa in the lungs of tolerant- and susceptible-to-hypoxia rats after 3, 6, and 24 hours of LPS administration. Data are presented as median (25%, 75%); the statistical significance of differences (*p*) between tolerant and susceptible rats is determined by the Mann-Whitney *U* test and between different time points after LPS injection by the Kruskal-Wallis method.

	Group	Control^1^	LPS			*p*<0.05
			3 h^2^	6 h^3^	24 h^4^	
Number of neutrophils in the interalveolar septa of the lungs, 25 000 *μ*m^2^	Tolerant	2.3	18.6	19.2	10.3	**0.005** ^**1-2**^
		(2.0–2.3)	(16.9–24.4)	(16.8–21.9)	(8.0–11.0)	**0.005** ^**1-3**^
	Susceptible	1.9	31.5	30.1	11.1	**0.002** ^**1-2**^
		(1.5–2.2)	(28.0–38.6)	(25.2–32.5)	(10.0–11.7)	**0.017** ^**1-3**^
	*p*	0.46	0.06	**0.04**	0.31	

**Table 2:** Subpopulation of lymphocytes in the peripheral blood of tolerant- (T) and susceptible- (S) to-hypoxia Wistar rats in 3, 6, and 24 hours after LPS injection. Data are presented as median (25%, 75%); the statistical significance of differences (*p*) between tolerant and susceptible rats is determined by the Mann-Whitney *U* test and between different time points after LPS injection by the Kruskal-Wallis method (*n* = 5 in each group).

Lymphocyte subpopulation, 10^6^/ml	Group	Control group^1^	LPS injection			*p*(*p*<0.05)
			3 h^2^	6 h^3^	24 h^4^	
Т-lymphocytes (CD3+)	T	3.5	2.7	1.7	6.0	**0.02** ^**2-4**^
		(2.7–4.3)	(2.1–2.8)	(1.2–2.6)	(5.4–6.2)	**0.04** ^**3-4**^
	S	7.0	2.3	1.9	4.6	**0.004** ^**1-3**^
		(4.8–7.7)	(2.2–3.2)	(1.7–2.2)	(3.9–5.2)	
	*p*	0.09	0.75	0.81	**0.03**	

Т-helper cells (CD3+CD4+)	T	2.2	1.1	0.4	2.7	**0.005** ^**3-4**^
		(1.6–2.4)	(1.0–1.1)	(0.3–0.5)	(1.9–3.1)	
	S	1.8	1.1	0.4	2.1	**0.02** ^**1-3**^
		(1.6–2.1)	(1.0–1.3)	(0.3–0.5)	(1.6–2.3)	**0.008** ^**3-4**^
	*p*	0.80	0.60	0.62	0.31	

Cytotoxic Т-lymphocytes (CD3+CD8+)	T	1.2	1.5	1.3	3.0	
		(1.0–1.8)	(1.0–1.7)	(0.8–2.0)	(2.5–4.1)	
	S	5.6	1.2	1.4	2.4	**0.04** ^**1-2**^
		(3.0–5.9)	(1.2–1.2)	(0.9–1.7)	(1.5–3.0)	
	*p*	**0.03**	0.75	0.62	0.19	

Regulatory Т-cells (CD4+CD25+ Foxp3+)	T	0.05	0.009	0.003	0.05	**0.04** ^**1-3**^
		(0.03–0.06)	(0.006–0.01)	(0.002–0.005)	(0.03–0.09)	**0.04** ^**2-4**^
						**0.003** ^**3-4**^
	S	0.05	0.01	0.006	0.03	**0.009** ^**1-3**^
		(0.02–0.05)	(0.007–0.01)	(0.004–0.006)	(0.01–0.05)	**0.04** ^**3-4**^
	*p*	0.81	0.75	0.14	0.24	

NK (CD314+)	T	0.8	0.7	1.4	2.6	**0.006** ^**2-4**^
		(0.8–1.2)	(0.6–1.2)	(1.3–2.1)	(2.1–3.5)	
	S	2.2	0.5	1.5	2.0	**0.01** ^**1-2**^
		(1.7–2.5)	(0.3–0.6)	(1.0–1.7)	(1.2–2.0)	**0.04** ^**2-4**^
	*p*	**0.03**	0.09	0.65	**0.04**	

В-lymphocytes (CD45+)	T	2.3	0.6	0.7	2.6	**0.009** ^**2-4**^
		(1.8–2.6)	(0.3–0.9)	(0.3–0.8)	(2.1–3.8)	**0.02** ^**3-4**^
	S	2.2	0.3	0.5	2.3	**0.04** ^**1-2**^
		(1.9–2.9)	(0.2–0.5)	(0.4–0.5)	(2.2–2.7)	**0.03** ^**2-4**^
	*p*	0.81	0.06	0.65	0.88	

**Table 3:** Subpopulation of lymphocytes in the peripheral blood of tolerant- (T) and susceptible- (S) to-hypoxia Wistar rats in 3, 6, and 24 hours after LPS injection. Data are presented as median (25%, 75%); the statistical significance of differences (*p*) between tolerant and susceptible rats is determined by the Mann-Whitney *U* test and between different time points after LPS injection by the Kruskal-Wallis method (*n* = 5 in each group).

Lymphocyte subpopulation	Group	Control group^1^	LPS injection			*p*(*p*<0.05)
			3 h^2^	6 h^3^	24 h^4^	
CD3+ Т-lymphocytes, % from lymphocytes	T	38.0	42.3	44.6	51.2	
		(28.4–45.9)	(35.1–51.8)	(25.3–45.2)	(40.0–57.9)	
	S	53.6	55.8	43.0	46.0	
		(44.2–54.1)	(48.5–56.4)	(37.9–45.9)	(40.2–51.5)	
	*p*	0.14	0.25	0.33	0.38	

CD3+CD4+ Т-helper cells, % from T-lymphocytes	T	57.5	46.1	20.3	42.2	**0.02** ^**1-3**^
		(49.0–66.3)	(42.0–51.5)	(16.4–21.2)	(29.8–50.4)	
	S	29.8	44.8	22.1	46.3	
		(23.2–34.4)	(34.3–61.0)	(21.3–23.8)	(43.1–50.5)	
	*p*	0.06	0.75	0.33	0.51	

CD3+CD8+ cytotoxic Т-lymphocytes, % from T-lymphocytes	T	40.8	52.7	76.6	54.6	**0.02** ^**1-3**^
		(31.8–49.3)	(47.7–57.2)	(74.0–82.6)	(46.3–67.7)	
	S	69.5	53.6	73.8	52.9	
		(63.5–76.1)	(37.8–62.9)	(69.4–76.8)	(46.2–54.6)	
	*p*	0.06	0.75	0.62	0.66	

CD4+CD25+ Foxp3+ regulatory Т-cells, % from T-helpers	T	2.1	1.0	0.7	2.2	
		(2.05–2.4)	(0.8–1.3)	(0.5–0.7)	(1.5–2.7)	
	S	1.8	1.2	1.1	1.9	
		(1.5–2.7)	(0.6–1.2)	(1.0–1.2)	(0.9–2.1)	
	*p*	0.62	0.92	0.39	0.34	

CD314+ NK, % from lymphocytes	T	8.6	15.6	29.6	27.3	**0.03** ^**1-3**^
		(8.3–12.7)	(11.6–19.9)	(26.0–37.1)	(15.8–30.7)	
	S	15.4	9.6	28.8	17.6	**0.003** ^**2-3**^
		(15.4–22.1)	(6.5–13.4)	(26.1–30.2)	(16.7–18.1)	
	*p*	0.33	0.14	0.65	0.56	

CD45+ В-lymphocytes, % from lymphocytes	T	25.3	14.5	9.3	20.8	**0.03** ^**1-3**^
		(19.4–27.8)	(5.9–15.3)	(5.6–11.5)	(19.5–22.0)	**0.03** ^**3-4**^
	S	17.1	6.4	10.1	25.2	**0.003** ^**2-4**^
		(13.2–20.2)	(4.9–8.6)	(8.9–10.5)	(25.2–27.7)	**0.02** ^**3-4**^
	*p*	0.09	0.33	0.88	**0.03**	

## Data Availability

The data used to support the findings of this study are available from the corresponding author upon request.

## Supplementary Materials

Supplementary Materials Figure S1: morphological changes in the spleen of tolerant- and susceptible-to-hypoxia male Wistar rats after 3, 6, and 24 hours of LPS administration. Hematoxylin and eosin staining. Original magnification: 100x. (A) Tolerant-to-hypoxia rat, control group; the white pulp is represented by the PALS and lymphoid follicles with germinal centers, and the marginal zone of lymphoid follicles is pronounced. (B) Susceptible-to-hypoxia rat, control group; lymphoid follicles of white pulp are large with wide germinal centers, and the marginal zone of lymphoid follicles is wide, represented by 12–14 rows of lymphocytes. (C) Tolerant-to-hypoxia rat, 3 h LPS; PALS is pronounced, large lymphoid follicle with a wide germinal center, and marginal zone is narrow. (D) Susceptible-to-hypoxia rat, 3 h LPS; PALS is pronounced, large lymphoid follicle with a wide germinal center, and marginal zone is narrow. (E) Tolerant-to-hypoxia rat, 6 h LPS; PALS is pronounced, large lymphoid follicle with a wide germinal center, and marginal zone is narrow. (F) Susceptible-to-hypoxia rat, 6 h LPS; devastation of the white pulp; PALS is not pronounced, lymphoid follicle with large germinal center, and marginal zone of lymphoid follicle is narrow, represented by 4-5 rows of lymphocytes. (G) Tolerant-to-hypoxia rat, 24 h LPS; pronounced devastation of white pulp; PALS is not pronounced, lymphoid follicle is small, without a germinal center, and marginal zone is represented by 5-6 rows of lymphocytes. (H) Susceptible-to-hypoxia rat, 24 h LPS; white pulp prevails, and PALS is wide, lymphoid follicles with wide marginal zones, represented by 9-10 rows of lymphocytes.
