# Supplementary material for: Morphological Characteristics of the Thymus and Spleen and the Subpopulation Composition of Lymphocytes in Peripheral Blood during Systemic Inflammatory Response in Male Rats with Different Resistance to Hypoxia
Source: Int J Inflam. 2019 Apr 1;2019:7584685. doi: 10.1155/2019/7584685 (PMC6463648; doi:10.1155/2019/7584685)
Supplement: Supplementary Materials — Figure S1: morphological changes in the spleen of tolerant- and susceptible-to-hypoxia male Wistar rats after 3, 6, and 24 hours of LPS administration. Hematoxylin and eosin staining. Original magnification: 100x. (A) Tolerant-to-hypoxia rat, control group; the white pulp is represented by the PALS and lymphoid follicles with germinal centers, and the marginal zone of lymphoid follicles is pronounced. (B) Susceptible-to-hypoxia rat, control group; lymphoid follicles of white pulp are large with wide germinal centers, and the marginal zone of lymphoid follicles is wide, represented by 12–14 rows of lymphocytes. (C) Tolerant-to-hypoxia rat, 3 h LPS; PALS is pronounced, large lymphoid follicle with a wide germinal center, and marginal zone is narrow. (D) Susceptible-to-hypoxia rat, 3 h LPS; PALS is pronounced, large lymphoid follicle with a wide germinal center, and marginal zone is narrow. (E) Tolerant-to-hypoxia rat, 6 h LPS; PALS is pronounced, large lymphoid follicle with a wide germinal center, and marginal zone is narrow. (F) Susceptible-to-hypoxia rat, 6 h LPS; devastation of the white pulp; PALS is not pronounced, lymphoid follicle with large germinal center, and marginal zone of lymphoid follicle is narrow, represented by 4-5 rows of lymphocytes. (G) Tolerant-to-hypoxia rat, 24 h LPS; pronounced devastation of white pulp; PALS is not pronounced, lymphoid follicle is small, without a germinal center, and marginal zone is represented by 5-6 rows of lymphocytes. (H) Susceptible-to-hypoxia rat, 24 h LPS; white pulp prevails, and PALS is wide, lymphoid follicles with wide marginal zones, represented by 9-10 rows of lymphocytes. [file 7584685.f1.pdf]

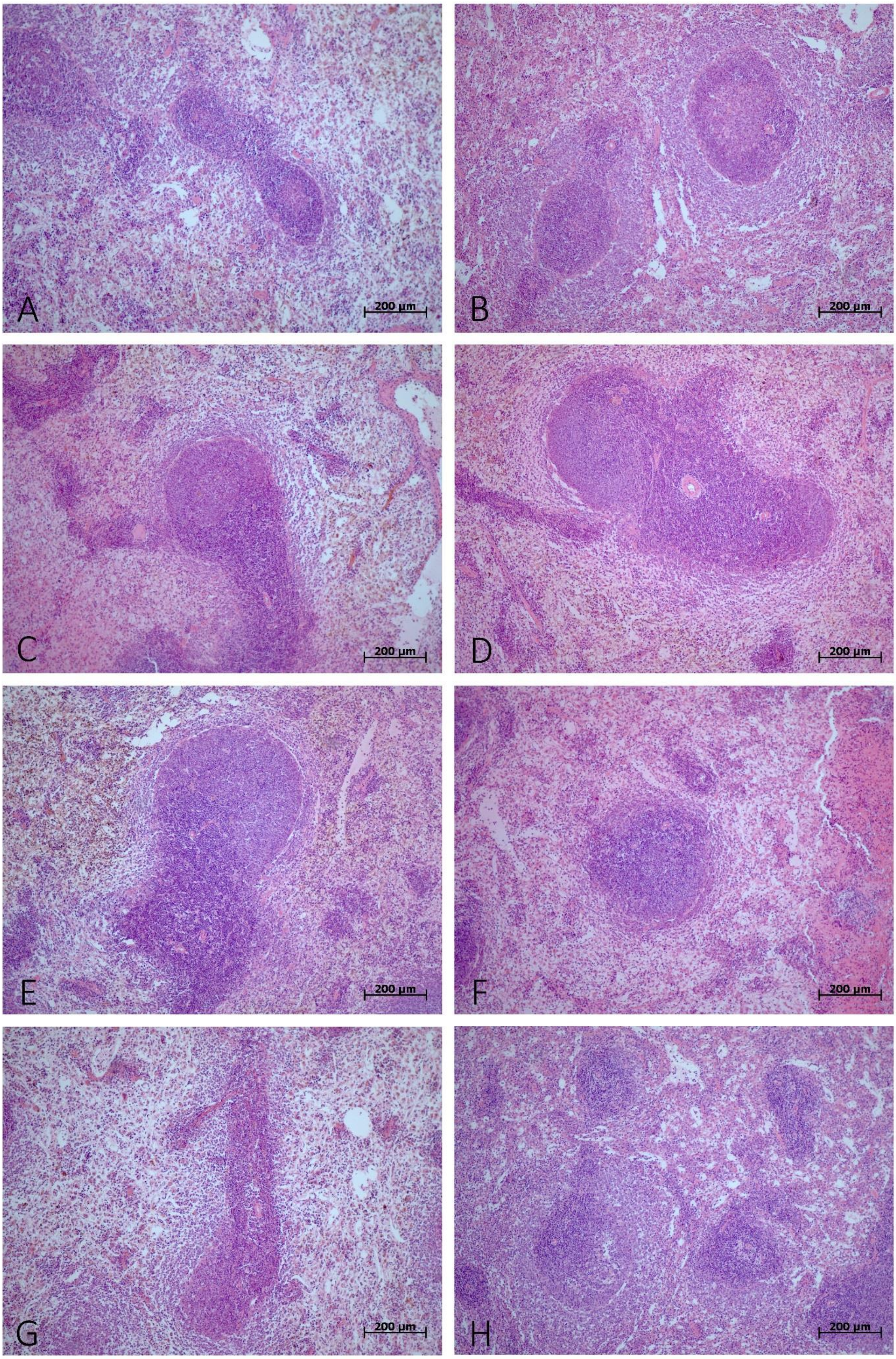
A
200 µm
B
200 µm
C
200 µm
D
200 µm
E
200 µm
F
200 µm
G
200 µm
H
200 µm

Figure S1: Morphological changes in the spleen of tolerant and susceptible to hypoxia male Wistar rats after 3, 6 and 24 hours of LPS administration. Haematoxylin and eosin staining. Original magnification 100x.

(A) Tolerant to hypoxia rat, control group – the white pulp is represented by the PALS and lymphoid follicles with germinal centers, the marginal zone of lymphoid follicles is pronounced.

(B) - Susceptible to hypoxia rat, control group – lymphoid follicles of white pulp are large with wide germinal centers, the marginal zone of lymphoid follicles is wide, represented by 12-14 rows of lymphocytes.

(C) Tolerant to hypoxia rat, 3h LPS – PALS is pronounced, large lymphoid follicle with a wide germinal center, marginal zone is narrow.

(D) Susceptible to hypoxia rat, 3h LPS – PALS is pronounced, large lymphoid follicle with a wide germinal center, marginal zone is narrow.

(E) Tolerant to hypoxia rat, 6h LPS – PALS is pronounced, large lymphoid follicle with a wide germinal center, marginal zone is narrow.

(F) Susceptible to hypoxia rat, 6h LPS – devastation of the white pulp, PALS is not pronounced, lymphoid follicle with large germinal center, marginal zone of lymphoid follicle is narrow, represented by 4-5 rows of lymphocytes.

(G) Tolerant to hypoxia rat, 24h LPS – pronounced devastation of white pulp, PALS is not pronounced, lymphoid follicle is small, without a germinal center, marginal zone is represented by 5-6 rows of lymphocytes.

(H) Susceptible to hypoxia rat, 24h LPS – white pulp prevails, PALS is wide, lymphoid follicles with wide marginal zones, represented by 9-10 rows of lymphocytes.
